# Development of two psychological experience questionnaires for screening violence-related mental health disorders of non-psychiatric inpatients

**DOI:** 10.1186/s12955-020-01399-9

**Published:** 2020-05-25

**Authors:** Yanjun Meng, Yuling Li, Hongbao Cao, Yong Xu, Binquan Wang

**Affiliations:** 1 Nursing College, Shanxi Medical University, 56 Xinjian South Road, Taiyuan, Shanxi 030001 People’s Republic of China; 2 Nursing College, Shanxi University of Chinese Medicine, Taiyuan, Shanxi People’s Republic of China; 3 School of Systems Biology, George Mason University (GMU), Fairfax, VA USA; 4 Department of Psychiatry, First Hospital of Shanxi Medical University, Taiyuan, Shanxi People’s Republic of China; 5 Department of Otolaryngology, Head and Neck Surgery, First Hospital of Shanxi Medical University, Taiyuan, Shanxi People’s Republic of China

**Keywords:** Psychological questionnaire, Violence, Anxiety, Depression, Suicidality, Paranoid personality disorder, Emotionally unstable personality disorder, Histrionic personality disorder

## Abstract

**Background:**

Increased violent events happen in the general hospitals in China and yet non-psychiatric departments do not have tools for violence-tendency screening.

**Methods:**

The current study developed and evaluated two Inpatient Psychological Experience Questionnaires (IPEQs) for the screening of violence-related six mental health disorders: (Inpatient Psychological Experience Questionnaire-1 (IPEQ-1): anxiety, depression and suicidality; Inpatient Psychological Experience Questionnaire-2 (IPEQ-2): paranoid personality disorder, emotionally unstable personality disorder and histrionic personality disorder). Two initial IPEQs (IPEQ-1: 37 items and IPEQ-2: 30 items) were developed and assessed by domain experts. Then 1210 inpatients were recruited and divided into three groups (160, 450 and 600 samples, respectively) for IPEQs item selection and evaluation. During the two-stage item selection, three statistical methods including Pearson’s correlation coefficient, exploratory factor analysis and item response theory were applied. For the item evaluation, Cronbach’s alpha coefficient, test-retest reliability, criterion-related validity and construct validity of the final questionnaires were measured.

**Results:**

Twelve items were selected for each IPEQs. Cronbach’s alpha coefficients were 0.91 and 0.78 for IPEQ-1 and IPEQ-2, respectively. Test-retest replication ratios were 0.95 and 0.87 for IPEQ-1 and IPEQ-2, respectively. Correlation coefficients between different disorders and their related-tools scores were [0.51, 0.44] and [0.40, 0.44] for IPEQ-1 and IPEQ-2, respectively and were significant (*P* < 0.01). Confirmatory factor analysis supported the validity of the final IPEQs (*P* < 0.05), and the model fit index met the criterion generally.

**Conclusion:**

The IPEQs developed in this study could be effective and easy-to-use tools for screening inpatients with violence-intendancy in non-psychosomatic departments.

## Background

In recent years, increased violent events were reported in the general hospitals of China [1, 2], which has threatened medical workers’ safety and even their lives, and has also jeopardized the relationship between medical worker and patient. A meta-analysis by Lu et al. 2018 covering 81,771 health-care professionals reported that the prevalence of workplace violence against health-care professionals was 62.4% [3]. Previous studies showed that non-psychiatric patients with mental health disorders were more likely to have a tendency to exhibit violence [4, 5]. For example, individuals with paranoid personality disorder (PPD), emotionally unstable personality disorder (EUPD) or histrionic personality disorder (HPD) had an increased tendency to suffer from comorbid psychological and physical problems and atypically exhibited a higher use of medical resources [6–8]. At the same time, there was a lack of flexibility in adapting to social, environmental and interpersonal communications [9]. The core phenomenological feature of the above personality disorders is the difficulty in emotional regulation [10]. Many individuals with such disorders have a biological susceptibility to experience emotional disturbances and are more likely to have a strong reaction to the experience. Their impulsive and angry behavior toward others, is regarded as a bad strategy they have adopted to alleviate or avoid intense negative emotional experiences [11]. PPD, EUPD and HPD are strongly associated with anxiety and depression, and the individuals with these disorders are more prone to suicide or interpersonal violence [12–14]. In this study, we hypothesized that individuals with demonstrated in the above-mentioned mental health disorders are more likely to present with a tendency to be violent.

However, the recognition rate of mental health disorders by non-psychiatric medical workers was very low in China [15]. The currently commonly used psychological-assessment tools, such as Kessler Psychological Distress Scale (K10) [16], Hospital Anxiety and Depression Scale (HADS) [17], were related to emotional disorders (e.g., anxiety and/or depression). These scales are rarely used in non-psychiatric routine clinical practice, and personality disorders are not assessed in non-psychiatric clinical setting, which may weaken the effectiveness of the mental health disorder screening. Patients with mental disorders are prone to suicidal ideation or suicidal behavior, while non-psychiatric medical workers pay little attention to this situation [18]. The “Huaxi Emotional-distress Index” (HEI) [19], a Chinese questionnaire, including 9 items for screening of depression and anxiety, was developed for use in non-psychiatric routine clinical practice of general hospitals. However, it failed to integrate personality disorders as screening items and thus had not reached high efficiency for patient selection of mental health disorders (e.g, anxiety and depression).

There were two main factors making it difficult to employ mental health disorders screening: first, the lack of necessary knowledge or skills in non-psychiatric departments for mental-health and related personality disorder assessment [20]; second, the complexity of existing assessment tools. To begin with, most of existing psychological-assessment tools were designed for the use by psychiatric-departments in hospitals. Non-psychiatric doctors and nurses usually do not gain enough professional-training in psychiatric assessment and care, and therefore it is difficult for them to use psychological-assessment tools. Furthermore, the existing assessment tools (e.g., HADS, Self-rating Anxiety Scale, Self-rating Depression Scale) usually contain too many items and patients may lose their attention during the assessment and give inaccurate answers [21]. Last but not least, the items and standard scores of the tools were specially designed for used in Westerner society. However, the translated version of the tools is rarely used in China hospitals due to the cultural and language barriers [22].

**Fig. 1:**
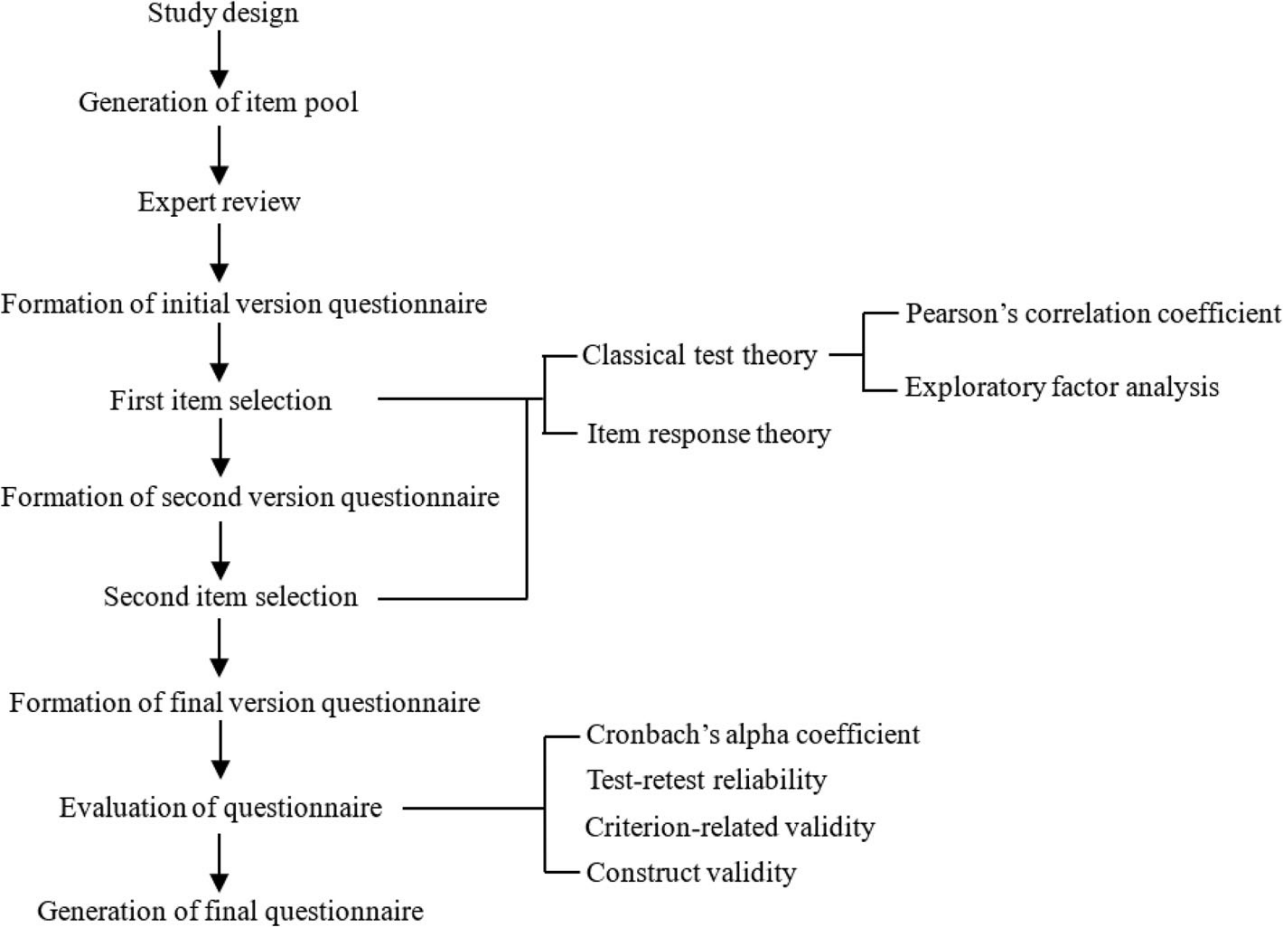
Study flow diagram

To address these issues, in this study we made efforts to learn from the design of existing assessment tools, and also integrate the clinical experience used in China, and develop the two questionnaires according that showed effective and easy-to-use in Chinese patients. IPEQ-1 is for the screening of anxiety, depression and suicidality; and IPEQ-2 is for the screening of PPD, EUPD and HPD. This article reported on the development of the initial questionnaire items, selection of the final questionnaire items, and evaluation of two new IPEQs. The entire study flow diagram was shown in Fig. 1.

## Materials and methods

### Participants

To develop and evaluate the proposed two IPEQs, we recruited 1210 participants, who were inpatients from different department of First Hospital of Shanxi Medical University (3A-grade general hospital, Taiyuan, Shanxi, China). All inpatients met the following inclusion criteria: (1) age ≥ 18 years, (2) being ability to complete the questionnaires independently, and (3) willing to participate in this study. Inpatients were excluded if they were unable to complete the questionnaire. The 1210 participants were separated into three groups for item selection and evaluation, with the sample size of 160, 450 and 600, respectively. The sample size of first-stage was calculated according to the ratio of subjects to items (the maximum number of items in the scale), which was suggested as 5:1 in the pre-investigation stage [23]; and the sample size to item ratio was set as 10:1 for the second and third stage [24]. The recruitment of these samples was as follows.

The first group was used for item pre-selection, which contains 160 inpatients recruited from July 2018 to August 2018. Specifically, 40 were from the psychiatric department, and 120 from non-psychiatric departments, including respiratory, neurology, traditional Chinese medicine, oncology and general surgery. The questionnaires with incomplete or biased answers were recognized as invalid and excluded. Finally, there were 149 inpatients completed IPEQ-1 and were included in the statistical analysis. For IPEQ-2, the number was 130.

The second group was used for the item-revaluation based on the second-stage item selection, which contains 450 inpatients, recruited from non-psychiatric departments in August and September, 2018, from several departments respiratory, neurology, traditional Chinese medicine, oncology, general surgery, nephrology, hematology, geriatrics, cardiology, gastroenterology, endocrinology, rheumatology, plastic surgery, urology, orthopedics, otolaryngology, neurosurgery and gynecology. For IPEQ-1, there were 423 valid questionnaires collected, and the number for IPEQ-2 was 419.

The third sample group was used for the evaluation of the proposed IPEQs. This group was composed of 600 non-psychiatric inpatients recruited from September 2018 to October 2018. There were 547 completed questionnaires collected for IPEQ-1and 523 for IPEQ-2.

We present participants demographics of three sample groups in Table 1.

**Table 1:** Participants demographics of three sample groups

Characteristic	First sample (IPEQ-1: *n* = 149; IPEQ-2: *n* = 130)		Second sample (IPEQ-1: *n* = 423; IPEQ-2: *n* = 419)		Third sample (IPEQ-1: *n* = 547; IPEQ-2: *n* = 523)	
	IPEQ-1 *n* (%)	IPEQ-2 *n* (%)	IPEQ-1 *n* (%)	IPEQ-2 *n* (%)	IPEQ-1 *n* (%)	IPEQ-2 *n* (%)
**Age**						
M ± SD	49.47 ± 15.16	49.55 ± 15.35	50.93 ± 17.84	50.88 ± 17.89	51.43 ± 16.92	51.34 ± 16.95
Missing	2 (1.34)	2 (1.54)	5 (1.18)	7 (1.67)	8 (1.46)	8 (1.53)
**Gender**						
Male	79 (53.02)	69 (53.08)	212 (50.12)	208 (49.64)	261 (47.71)	250 (47.80)
Female	66 (44.30)	57 (43.85)	207 (48.94)	207 (49.40)	282 (51.55)	269 (51.43)
Missing	4 (2.68)	4 (3.08)	4 (0.95)	4 (0.95)	4 (0.73)	4 (0.76)
**Marital status**						
Single	16 (10.74)	14 (10.77)	48 (11.35)	48 (11.46)	54 (9.87)	51 (9.75)
Married/cohabitating	126 (84.56)	109 (83.85)	345 (81.56)	342 (81.62)	469 (85.74)	449 (85.85)
Divorced/separated/widowed	3 (2.01)	3 (2.31)	24 (5.67)	23 (5.49)	15 (2.74)	15 (2.87)
Missing	4 (2.68)	4 (3.08)	6 (1.42)	6 (1.43)	9 (1.65)	8 (1.53)
**Education level**						
Junior school or less	56 (37.58)	48 (36.92)	179 (42.32)	178 (42.48)	218 (39.85)	206 (39.39)
Senior/polytechnic school	40 (26.85)	32 (24.62)	114 (26.95)	111 (26.49)	161 (29.43)	155 (29.64)
College	37 (24.83)	34 (26.15)	112 (26.48)	112 (26.73)	134 (24.50)	130 (24.86)
Graduate school	8 (5.37)	8 (6.15)	9 (2.13)	9 (2.15)	18 (3.29)	17 (3.25)
Missing	8 (5.37)	8 (6.15)	9 (2.13)	9 (2.15)	16 (2.93)	15 (2.87)

### Item generation of questionnaires

**Fig. 2:**
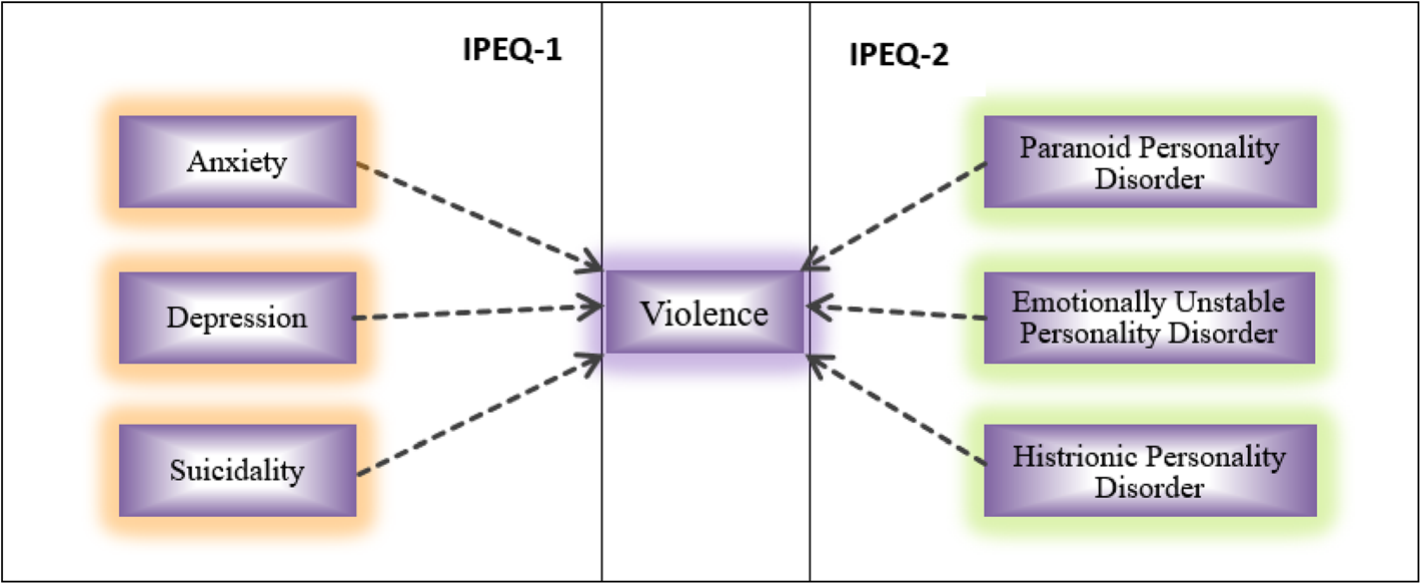
Study design and the network between violence and six mental health disorders**.** IPEQ-1: Questionnaire for screening mental health disorders related to anxiety, depression and suicidality; IPEQ-2: Questionnaire for screening mental health disorders related to paranoid personality disorder, emotionally unstable personality disorder and histrionic personality disorder

From a literature review, we designed two questionnaires for the screening of violence-related six mental health disorders: IPEQ-1: screening anxiety, depression and suicidality; IPEQ-2: screening PPD, EUPD and HPD. (Fig. 2).

The item pool of IPEQs were generated according to International Statistical Classification of Diseases and Related Health Problems 10th Revision (ICD-10) [25], which is used to provide official criteria in clinical diagnosis. ICD-10 codes: anxiety: F41.1, depression: F32, suicidality: F32 (e); paranoid personality disorder: F60.0, emotionally unstable personality disorder: F60.3, histrionic personality disorder: F60.4. And we drafted the items by reference of existing self-completed screening scales. The main reference in this study included the Symptom Checklist-90 (SCL-90) [26], HADS [17], Self-rating Anxiety Scale (SAS) [27], Beck Anxiety Inventory (BAI) [28], Generalized Anxiety Disorder-7 (GAD-7) [29], Self-rating Depression Scale (SDS) [30], Beck Depression Inventory (BDI) [31], Patient Health Questionnaire-9 items (PHQ-9) [32], HEI [19], Beck Scale for Suicide Ideation-Chinese Version (BSI-CV) [33], and Personality Diagnostic Questionnaire-4+ (PDQ-4+) [34].

The candidate item of IPEQs were reviewed by a panel of 16 senior experts that consisted of professors, clinicians and researchers in the field of psychology and psychiatry from 5 cities in China (Shanxi, Beijing, Hebei, Zhejiang and Jiangsu). These experts had more than 10-year experiences in the field. They rated each item in the pool as “strongly approved,” “revise slightly,” “revise moderately,” “revise entirely or delete,” or “delete”; and put forward specific suggestions for revision.

Initially, 37 and 30 items were selected for IPEQ-1 and IPEQ-2, respectively (Table 2 and Table 3), which were evaluated following an approach described in the following sections.

**Table 2:** The results of PCC, EFA and IRT obtained from the 37 (initial version) and 18 (second version) items of IPEQ-1

Disorder	Item	PCC	EFA	IRT					PCC	EFA	IRT				
		Correlation coefficient	Factor loading	*a*	*b*_*1*_	*b*_*2*_	*b*_*3*_	*b*_*4*_	Correlation coefficient	Factor loading	*a*	*b*_*1*_	*b*_*2*_	*b*_*3*_	*b*_*4*_
Anxiety	1. Felt worried or nervous?	0.76^**^	0.69	2.03	−1.29	−0.26	0.87	2.00	0.79^**^	0.81	0.23	−3.81	1.68	10.38	28.72
	2. Felt afraid for no reason?	0.69^**^	0.76	1.68	− 0.54	0.39	1.52	3.21	0.79^**^	0.80	0.28	−4.74	−1.38	1.35	4.74
	3. Felt uneasy?	0.77^**^	0.74	2.36	−1.00	−0.10	1.09	2.79	0.86^**^	0.80	0.22	−6.87	−2.45	1.24	5.62
	4. Felt too unstable to calm down?	0.73^**^	0.78	2.00	−0.83	0.04	1.15	2.95	0.82^**^	0.73	1.42	−0.78	0.70	1.95	3.05
	5. Felt dizzy and had a headache?	0.61^**^	0.42	1.32	−0.73	0.52	1.35	3.03							
	6. Felt restless or had shaking hands and feet?	0.71^**^	0.47	2.14	−0.41	0.39	1.18	1.89							
	7. Felt chest tightness and shortness of breath?	0.71^**^	0.79	1.26	−0.67	0.32	1.21	3.12							
	8. Felt heart beating fast?	0.76^**^	0.77	1.60	−0.52	0.61	1.18	3.34							
	9. Felt muscle tension?	0.79^**^	0.65	2.04	−0.21	0.54	1.43	2.94							
	10. Felt pain?	0.71^**^	0.74	1.19	−0.50	0.61	2.10	3.29							
	11. Felt uncomfortable but didn’t know why?	0.74^**^	0.55	1.80	−1.21	0.09	1.20	3.11	0.72^**^	0.53	1.55	0.04	1.17	2.31	3.35
	12. Felt like you were dying?	0.65^**^	0.56	3.18	−0.14	0.36	0.81	1.32							
Depression	13. Felt unhappy?	0.77^**^	0.61	3.20	−0.99	−0.19	0.54	1.43	0.71^**^	0.64	1.99	−0.30	1.00	1.89	2.88
	14. Felt so depressed that nothing could make you happy?	0.79^**^	0.53	3.38	−0.79	− 0.09	0.41	1.11	0.71^**^	0.59	2.07	−0.18	1.01	2.09	3.48
	15. Felt depressed and hopeless?	0.83^**^	0.47	4.02	−0.60	0.11	0.45	1.19							
	16. Felt less interested in everyday activities?	0.80^**^	0.72	3.80	−1.02	−0.14	0.39	1.25	0.78^**^	0.73	2.08	−0.46	0.76	1.62	2.57
	17. Felt uninterested in any part of your daily routine?	0.77^**^	0.77	3.52	−0.96	−0.11	0.45	1.27	0.85^**^	0.79	2.14	−0.70	0.70	1.85	2.65
	18. Felt like you were not interested in doing anything?	0.80^**^	0.65	3.77	−0.86	−0.12	0.29	1.23	0.83^**^	0.75	2.42	−0.19	0.90	1.70	2.62
	19. Felt unable to proceed with daily tasks?	0.78^**^	0.63	3.03	−1.21	−0.32	0.55	2.65	0.80^**^	0.80	2.41	−0.55	0.70	1.82	2.70
	20. Felt tired and listless?	0.70^**^	0.53	2.09	−1.55	−0.29	0.71	1.65	0.75^**^	0.72	3.18	−0.39	0.68	1.55	2.25
	21. Felt loss of appetite or were unable to appreciate the deliciousness of food?	0.67^**^	0.55	2.10	−0.86	0.03	1.02	1.61							
	22. Had insomnia?	0.62^**^	0.45	1.65	−0.89	0.24	1.23	2.45							
	23. Felt easily angered?	0.64^**^	0.48	1.72	−1.00	0.15	0.81	1.74							
	24. Felt like you were causing a lot of trouble to others?	0.77^**^	0.60	2.62	−1.28	−0.28	0.24	0.92	0.72^**^	0.60	3.03	−0.26	0.79	1.53	2.11
	25. Felt like a useless person?	0.83^**^	0.68	3.60	−0.71	−0.12	0.23	1.26	0.76^**^	0.62	2.07	−0.82	0.55	1.43	2.36
	26. Felt inferior to everyone else?	0.76^**^	0.75	2.56	−0.63	0.01	0.65	1.37							
	27. Felt that your brain response was slow or your memory was poor?	0.68^**^	0.73	1.90	−1.40	−0.24	0.72	2.15							
	28. Felt unable to concentrate?	0.79^**^	0.77	2.66	−1.00	−0.15	0.61	2.70							
	29. Frequently drank alcohol or took addictive substances?	0.24^**^	0.71	0.53	2.72	5.19	7.25	12.51							
	30. Felt like crying for no reason?	0.62^**^	0.48	1.96	−0.19	0.69	1.71	2.07							
	31. Felt hyposexual?	0.55^**^	0.57	1.44	−0.76	0.19	1.12	1.88							
	32. Felt as though the day were passing as if it were a year?	0.78^**^	0.57	3.81	−0.50	0.08	0.71	1.26							
	33. Felt purposeless in life?	0.85^**^	0.65	4.68	−0.53	0.05	0.58	1.12	0.86^**^	0.62	1.84	−0.99	0.44	1.29	2.46
Suicidality	34. Felt life was meaningless?	0.87^**^	0.56	5.08	−0.37	0.24	0.57	1.07							
	35. Felt death would be a release?	0.92^**^	0.62	3.68	−0.18	0.42	0.66	1.25	0.89^**^	0.80	1.73	−0.67	0.58	1.63	2.41
	36. Had thoughts of ending your life?	0.90^**^	0.74	2.91	0.12	0.69	1.47	2.62	0.86^**^	0.87	2.25	−0.05	0.96	1.90	2.46
	37. Self-harmed or performed suicidal behavior?	0.78^**^	0.79	2.23	0.49	0.94	1.26	1.59	0.74^**^	0.80	2.51	0.16	1.09	1.79	3.12

^**^. Correlation is significant at the 0.01 level (2-tailed)

**Table 3:** The results of PCC, EFA and IRT obtained from the 30 (initial version) and 21 (second version) items of IPEQ-2

Disorder	Item	PCC	EFA	IRT		PCC	EFA	IRT	
		Correlation coefficient	Factor loadings	*a*	*b*	Correlation coefficient	Factor loadings	*a*	*b*
Paranoid personality disorder	1. You care too much about other people’s unfriendly attitudes, speech or behavior?	0.53^**^	0.79	0.82	0.57				
	2. You resent the harm, insult or contempt others have inflicted on you?	0.54^**^	0.75	1.08	0.58				
	3. You are vigilant in dealing with people to prevent them from using or harming you?	0.67^**^	0.72	1.60	0.18	0.59^**^	0.61	1.07	1.22
	4. You think that others are hostile or contemptuous of you?	0.81^**^	0.69	2.71	−0.04	0.52^**^	0.60	2.07	1.74
	5. Conflicts arise between you and people because of trifles?	0.83^**^	0.67	4.02	0.11	0.57^**^	0.53	1.70	1.60
	6. You suspect your spouse (or partner) of being unfaithful to you?	0.77^**^	0.60	3.24	0.23	0.50^**^	0.43	1.39	2.30
	7. You’re always unsatisfied with what people around you are doing?	0.76^**^	0.53	2.70	0.17	0.66^**^	0.53	1.64	1.03
	8. You think that others will take advantage of you or try to deceive you?	0.83^**^	0.69	5.16	0.14	0.71^**^	0.67	2.48	1.39
	9. You hesitate to trust others or cannot trust others?	0.71^**^	0.57	2.51	0.23	0.64^**^	0.54	1.63	1.27
	10. It’s uncomfortable to see others work and live better than you?	0.73^**^	0.42	3.08	0.17				
	11. You think you are right and others are wrong?	0.68^**^	0.49	2.20	0.22				
	12. It’s not easy to forgive others’ mistakes?	0.77^**^	0.65	2.72	0.27	0.56^**^	0.45	1.25	1.50
Emotionally unstable personality disorder	13. A little thing can cause great emotional fluctuations?	0.50^**^	0.70	1.02	0.40				
	14. You talk and do things without thinking?	0.77^**^	0.76	2.09	−0.03	0.62^**^	0.65	1.28	1.10
	15. It’s easy to do things recklessly and fail to control your actions well?	0.74^**^	0.77	1.67	0.15	0.70^**^	0.71	1.76	1.04
	16. It is difficult to control yourself and not get angry?	0.45^**^	0.79	0.56	1.24				
	17. You often do something reckless?	0.83^**^	0.56	3.72	0.27	0.66^**^	0.70	2.18	1.40
	18. It’s hard to control your anger or even refrain from hurting people?	0.76^**^	0.64	5.86	0.08				
	19. You can’t handle relationships with others well?	0.77^**^	0.51	2.79	0.10	0.65^**^	0.54	1.96	1.21
	20. You don’t know what kind of person you want to be?	0.70^**^	0.59	1.76	0.19	0.66^**^	0.47	1.57	0.81
	21. You feel empty and unable to live, study or work well?	0.71^**^	0.45	2.42	0.39				
Histrionic personality disorder	22. You exaggerate when expressing emotions?	0.79^**^	0.64	3.81	0.13				
	23. Talking to people is as vivid as acting?	0.82^**^	0.59	3.41	0.11	0.60^**^	0.54	1.02	1.66
	24. You are susceptible to others or circumstances changing your thoughts and behavior?	0.53^**^	0.68	1.44	0.52	0.57^**^	0.34	1.08	0.85
	25. People around you feel that you are not calm enough?	0.63^**^	0.54	1.73	0.25	0.66^**^	0.65	1.47	1.24
	26. You like to pursue new and exciting things and participate in various social activities?	0.67^**^	0.61	1.55	0.16	0.65^**^	0.56	0.81	1.00
	27. In order to attract people’s attention, you may pretend to surprise at small matters?	0.90^**^	0.62	8.61	0.08	0.64^**^	0.62	1.96	1.72
	28. You consider yourself more attractive than others?	0.84^**^	0.74	3.87	0.11	0.67^**^	0.72	1.05	1.84
	29. You draw the attention of others by your clothes or by certain behaviors?	0.81^**^	0.73	2.61	0.15	0.75^**^	0.77	2.09	1.60
	30. You care about your appearance?	0.68^**^	0.73	1.64	0.18	0.63^**^	0.53	1.10	1.10

^**^. Correlation is significant at the 0.01 level (2-tailed)

### Item selection of questionnaires

A two-stage item selection has been conducted with the first two aforementioned groups. The survey data were analyzed by employing both classical test theory (CTT) (including Pearson’s correlation coefficient (PCC) and exploratory factor analysis (EFA)) and item response theory (IRT).

In the first stage of item selection, the participants in the first sample group (*n* = 160) were asked to answer all 67 questions of the two initial questionnaires (37 questions in IPEQ-1, and 30 in IPEQ-2; see Table 2 and Table 3, respectively). We used PCC to test the strength of relation between the disorder types and their related question-items within each questionnaire. A question was recognized as valid if it demonstrated strong correlation with its corresponding disorder (PCC > 0.40 and *P* < 0.05), otherwise, it was excluded [35]. Then EFA was used to evaluate the overall significance of a question to its corresponding disorder and to the questionnaire as a whole. A principal-component method with varimax rotation was performed. The number of factors were decided according to the study design, the eigenvalue and the contribution ratio of cumulative variance. Items with low factor loading for its corresponding disorder (factor loading < 0.40) or cross-loading on two or more disorders were excluded [36].

In addition, IRT was used to test the relationship between subjects’ disorders and their responses to questions by the discrimination (*a*) of each item [37, 38]. For IPEQ-1, Samejima’s hierarchical response model was employed as the questionnaire was in the form of five-point Likert; for IPEQ-2, a two-parameter logistic model was employed as the questionnaire accepted binary answers. An item was excluded if it did not represent its corresponding disorder (*a* < 0.40) [39]. Based on the analysis of the results, 18 and 21 items were selected for IPEQ-1 and IPEQ-2, respectively. (Table 2 and Table 3).

For the second stage of item selection, the second inpatient group (*n* = 450) were required to answer the 18-item version of IPEQ-1 and 21-item version of IPEQ-2 from the first-stage item selection. The survey data were analyzed following the same statistical workflow (CTT and IRT) as used in the first stage item selection. IPEQs aimed to include the items that were not only efficient but also brief. The selection of an item was based on its consistency with primary symptoms of its corresponding disorder in ICD-10 and its statistical significance to the questionnaire. After the two-stage item selection, 12 questions were selected for both IPEQ-1 and IPEQ-2, which were used as the final version IPEQs (Supplementary Table 1 and Supplementary Table 2).

The total score of final version IPEQ-1 ranged among 0 ~ 48, with higher scores indicate greater severity of emotional distress and violence-tendency. The following cutoff scores have been suggested: ≤11 = normal, 12 ~ 16 = mild, 17 ~ 21 = moderate, ≥22 = severe. The total score of the final version IPEQ-2 range was 0 ~ 12, with higher scores indicate an increased likelihood of having a personality disorder and violence-tendency. The score range of each personality disorder was 0 ~ 4, the cutoff score of 3 or greater was adopted for screening of each personality disorder.

### Evaluation of final questionnaires

#### Reliability

The third group (*n* = 600) were used to evaluate the reliability and validity of both questionnaires. The internal disorder-specificity were measured by using Cronbach’s alpha coefficient (threshold ≥0.50) [40]. The stability of the questionnaires was tested by a test-retest process: four days after the initial test, 62 patients out of the 600 inpatients were randomly selected to answer two IPEQs one more time and the replication of the answers were calculated and used to evaluate the stability of the questionnaires. The acceptable reliability was set as correlation coefficient ≥ 0.70 and *P* < 0.05 [41].

#### Criterion-related validity

The efficacy of the propose questionnaires was evaluated by measuring the consistency between the results of using the proposed IPEQs and the three commonly used existing psychometric assessment-tools: 238 out of 600 were randomly picked to completed self-evaluation using GAD-7 [29], PHQ-9 [32], and PDQ-4+ [34]. Survey data were collected and compared with that from the two proposed IPEQs [42].

The GAD-7 was a 7-item anxiety scale, that score ranged from 0 to 21. Cronbach’s alpha was 0.92 for the GAD-7. The PHQ-9 was a 9-item depression questionnaire, which the ninth item pertains to self-harm. The PHQ-9 score ranged from 0 to 27 and Cronbach’s alpha was reported by developers to be 0.89 and 0.86. The PDQ-4+ was a 107-item questionnaire, designed to assess the 12 personality disorders (e.g., paranoid personality disorder, borderline personality disorder of emotionally instable personality disorder, histrionic personality disorder, etc.). The cutoff score of 4 or 5 was for screening of personality disorders. Cronbach’s alpha was 0.51 ~ 0.74 for the PDQ-4 + .

#### Construct validity

The constructional validity was measured by evaluating the relation between each item and it corresponding disorder using confirmatory factor analysis (CFA), with criteria set as: 1), factor loading > 0.50 and *P* < 0.05; 2), root mean square error of approximation (RMSEA) < 0.08; 3), goodness-of-fit index (GFI) > 0.90; 4), adjusted goodness of fit index (AGFI) > 0.90; 5), normed fit index (NFI) > 0.90; 6), non-normed fit index (NNFI) > 0.90; and 7), comparative fit index (CFI) > 0.90 [43].

#### Feasibility analysis

The feasibility of the IPEQs was evaluated by examining completion rate and completion time. When the questionnaire can be understood and completed by participants easily, it is considered acceptable feasibility.

The statistical analysis was conducted by using SPSS software version 20.0, MULTILOG software version 7.03 and LISREL software version 8.70.

## Results

### Item selection results

The results of the statistical analysis for the two-stage item selection were provided in Table 2 and Table 3, for IPEQ-1 and IPEQ-2, respectively. According to the selection criteria, 19 items from IPEQ-1 (37-item) and 9 items from IPEQ-2 (30-item) were excluded in the first stage of item selection, then items 6 and 9 were excluded for IPEQ-1 and IPEQ-2, respectively (Supplementary Fig. 1 and Supplementary Fig. 2).

Some items were to be excluded based on statistical results. For example, item 29 in IPEQ-1 was excluded, because the correlation coefficient was < 0.40. Item 12 in IPEQ-1 was excluded, because it was showed weak correlation with its corresponding disorder in PCC. The correlation coefficient between item 12 and suicidality was 0.71, which was higher than correlation with anxiety (coefficient = 0.65). Item 18 in IPEQ-2 was excluded, because it was also demonstrated weak correlation with its corresponding disorder in PCC. The correlation coefficient between item 18 and HPD was 0.78, which was higher than correlation with EUPD (coefficient = 0.76). Item 23, item 30 and item 32 in IPEQ-1 and item 1, item 2, item 13 and item 16 in IPEQ-2 were excluded, because there was no correlation between these items and their corresponding disorder in EFA.

Some items were to be excluded based on its consistency with primary symptoms of its corresponding disorder in ICD-10 in order to achieve two brief questionnaires. For example, item 5 was excluded because it was about physical symptoms. Item 15 was excluded because it had a lower factor loading compared to other items in depression in EFA, and its language expression was close to the item of suicidality.

Eventually, for each of two IPEQs, 12 items passed the selection scrutiny and were used as final versions of the questionnaires. As seen in Supplementary Table 1 and Supplementary Table 2, within IPEQ-1, items 1 to 4 were related to anxiety, 5 to 8 to depression and 9 to12 to suicidality; within IPEQ-2, items 1 to 4 were related to PPD, 5 to 8 to EUPD and 9 to 12 to HPD.

### Evaluation results of final IPEQs

#### Reliability

Cronbach’s alpha coefficient analysis indicated that items within both questionnaires demonstrated sufficient internal disorder-specificity (> 0.50). Test-retest reliability analysis indicated that they showed strong stability (correlation coefficient > 0.70 and *P* < 0.01). (Table 4).

**Table 4:** Cronbach’s alpha coefficient and correlation coefficient of test-retest reliability obtained from two IPEQs

Questionnaire	Disorder	The number of items	Cronbach’s alpha coefficient	Correlation coefficient
IPEQ-1	Anxiety	4	0.85	0.94^**^
	Depression	4	0.86	0.91^**^
	Suicidality	4	0.83	0.86^**^
	total	12	0.91	0.95^**^
IPEQ-2	Paranoid personality disorder	4	0.60	0.81^**^
	Emotionally unstable personality disorder	4	0.64	0.79^**^
	Histrionic personality disorder	4	0.63	0.82^**^
	total	12	0.78	0.87^**^

^**^. Correlation is significant at the 0.01 level (2-tailed)

#### Criterion-related validity

The correlation coefficients between IPEQs and GAD-7, PHQ-9, PDQ-4+ were significant (*P* < 0.01), indicated acceptable criterion-related validity. (Table 5).

**Table 5:** Correlation coefficients of criterion-related validity obtained between IPEQs and GAD-7, PHQ-9, PDQ-4+

Questionnaire	Disorder	GAD-7	PHQ-9	PDQ-4+		
				Paranoid personality disorder	Emotionally unstable personality disorder	Histrionic personality disorder
IPEQ-1	Anxiety	0.51^**^				
	Depression and Suicidality		0.44^**^			
IPEQ-2	Paranoid personality disorder			0.40^**^		
	Emotionally unstable personality disorder				0.41^**^	
	Histrionic personality disorder					0.44^**^

^**^. Correlation is significant at the 0.01 level (2-tailed)

#### Construct validity

As seen in Table 6, the CFA analysis indicated good construct validity in IPEQ-1. Factor loadings for each of the 12 items were above 0.50 (*P* < 0.05). The values of GFI, NFI, NNFI and CFI were met the acceptance criteria.

**Table 6:** CFA obtained from the 12 items of IPEQ-1

Disorder	Item	Factor loading	Standard error	*t*	R^2^	Error variance
Anxiety	1. Felt worried or nervous?	0.70	0.03	17.92^*^	0.49	0.37
	2. Felt afraid for no reason?	0.71	0.03	18.22^*^	0.51	0.26
	3. Felt uneasy?	0.83	0.03	22.69^*^	0.69	0.19
	4. Felt too unstable to calm down?	0.81	0.03	21.70^*^	0.65	0.21
Depression	5. Felt so depressed that nothing could make you happy?	0.83	0.03	22.89^*^	0.69	0.22
	6. Felt uninterested in any part of your daily routine?	0.83	0.03	23.06^*^	0.70	0.23
	7. Felt unable to proceed with daily tasks?	0.73	0.04	18.93^*^	0.53	0.43
	8. Felt tired and listless?	0.71	0.04	18.42^*^	0.51	0.46
Suicidality	9. Felt purposeless in life?	0.81	0.03	21.72^*^	0.66	0.19
	10. Felt death would be a release?	0.79	0.02	20.95^*^	0.63	0.14
	11. Had thoughts of ending your life?	0.78	0.02	20.58^*^	0.61	0.11
	12. Self-harmed or performed suicidal behavior?	0.58	0.02	14.04^*^	0.34	0.15
Fit index	RMSEA = 0.11	NFI = 0.96				
	GFI = 0.90	NNFI = 0.95				
	AGFI = 0.85	CFI = 0.96				

^*^P < 0.05

As seen in Table 7, the CFA analysis indicated good construct validity in IPEQ-2. Factor loadings for each of the 12 items were above 0.50 (*P* < 0.05), except for items 1, 9 and 12. However, these three items were recommended for retention by the results of CTT and IRT analysis. The values of RMSEA, GFI, AGFI, NFI, NNFI and CFI were met the acceptance criteria.

**Table 7:** CFA obtained from the 12 items of IPEQ-2

Disorder	Item	Factor loading	Standard error	*t*	R^2^	Error variance
Paranoid personality disorder	1. You are vigilant in dealing with people to prevent them from using or harming you?	0.47	0.02	9.52^*^	0.22	0.14
	2. You’re always unsatisfied with what people around you are doing?	0.52	0.02	10.61^*^	0.27	0.11
	3. You think that others will take advantage of you or try to deceive you?	0.59	0.02	12.32^*^	0.35	0.06
	4. You hesitate to trust others or cannot trust others?	0.52	0.02	10.75^*^	0.27	0.10
Emotionally unstable personality disorder	5. You talk and do things without thinking?	0.51	0.02	10.22^*^	0.26	0.12
	6. It’s easy to do things recklessly and fail to control your actions well?	0.59	0.02	11.95^*^	0.35	0.11
	7. You often do something reckless?	0.51	0.02	10.24^*^	0.26	0.07
	8. You can’t handle relationships with others well?	0.55	0.02	11.11^*^	0.30	0.10
Histrionic personality disorder	9. In order to attract people’s attention, you may pretend to surprise at small matters?	0.44	0.01	8.90^*^	0.20	0.07
	10. You consider yourself more attractive than others?	0.57	0.02	11.53^*^	0.32	0.08
	11. You draw the attention of others by your clothes or by certain behaviors?	0.58	0.02	11.87^*^	0.34	0.06
	12. You care about your appearance?	0.46	0.02	9.34^*^	0.21	0.14
Fit index	RMSEA = 0.05	NFI = 0.93				
	GFI = 0.97	NNFI = 0.95				
	AGFI = 0.95	CFI = 0.96				

^*^P < 0.05

#### Feasibility analysis

The completion rate for IPEQ-1 and IPEQ-2 were 91.17% (547/600) and 87.17% (523/600), respectively. The average completion time were 3.19 ± 3.09 min and 3.71 ± 3.39 min, respectively.

## Discussion

China legislated against violence to medical workers [44]. Unfortunately, increased violence events are still occurring in the general hospital in China, and doctors and nurses are usually caught un-prepared [45]. 2015–2016 Chinese physician-patient relationship blue book showed that in analyzing the characteristic of perpetrators showing in violence to medical workers, impulsiveness took 65.05%, especially in injury event and killing event, the proportions reached 78.60 and 84.21% respectively [46]. Some patients with certain mental health disorders are more likely to occur impulsiveness and violence [4, 5]. Therefore, by using clinical symptoms or personality traits associated with violence-related mental health disorders, patients with violent tendencies would be screened. However, the recognition rate of mental health disorders was very low. Existing psychiatric assessment tools are too complicated for non-psychiatric department to use. Therefore, it is of urgent need to develop an easy-to-use and yet effective screening tool for certain mental health disorders screening. In this study, we developed and evaluated two IPEQs, which were designed for the screening of six mental health disorders. We hypothesize that a patient (not necessarily with mental health disorders) with symptoms of these mental health disorders would present a higher risk of becoming violent.

Compared with three commonly used tools for psychological-assessment tools, which are usually used by professionals in psychiatric departments, our IPEQs demonstrated strong consistence(*P* < 0.01). These three tools are rarely directly used by non-psychiatric departments for multiple reasons. First, these tools were specifically designed for the use by psychiatric department and non-psychiatric doctors and nurses usually lack professional training for the effective use of those tools. Second, these tools usually contain too many question items and inpatients usually are short of patience to answer [47]. For example, both items in GAD-7 and PHQ-9 were combined to reach a total of 16 questions to cover only anxiety and depression. In PDQ-4+, 27 items were employed to cover three personality disorders. Thus, an inpatient has to answer 43 questions in total to test 6 mental health disorders. In comparison, each of our IPEQs contains only 12 items and showed similar efficacy in the screening of 6 mental health disorders. Doctors and nurses in the non-psychiatric department could easy to use our proposed IPEQs.

These two IPEQs were developed based on the ICD-10, experts’ opinions and relevant scale reviews. Three statistical methods (PCC, EFA and IRT) were considered in the two-stage item selection. Moreover, our statistical analysis showed that the items within each IPEQ showed high internal consistency (The Cronbach’ s alpha coefficients were 0.91 and 0.78 for IPEQ-1 and IPEQ-2, respectively) and strong stability (Test-retest replication ratios were 0.95 and 0.87 for IPEQ-1 and IPEQ-2, respectively). The final IPEQs also well-matched with the original IPEQs in terms of structure (Factor loadings were > 0.50 and *P* < 0.05). These results showed, as simple as our IPEQs (12 questions only), it is effective in screening the proposed six mental health disorders, which were implicated to be associated with violence tendency [40, 41, 43].

In our study, normal subjects were suggested P25 (cut-off score of 12) for IPEQ-1 cut-off by the percentile of the questionnaire total score, according to literature reports [48, 49]. ICD-10 showed that for diagnosing most of the subtypes’ personality disorders, clear evidence is usually required of the presence of at least three of the traits or behaviors given in the clinical description. So, IPEQ-2 cut-off score was set as 3 in our study. Our investigation research showed that the incidence of emotional distress among non-psychiatric inpatients was 23.81%. The incidence of paranoid personality disorder was 10.89%, emotionally unstable personality disorder was 11.87% and histrionic personality disorder was 9.05%.

Medical workers have traditionally assessed violence risk on an individual basis when it has happened. It is important to screen for potentially violent inpatients according to the underlying psychiatric motive, such as impulsiveness, through relatively accurate predicable tool. Previous studies showed that there were positive association between six mental disorders (anxiety, depression, suicidality, PPD, EUPD and HPD) and violence [12–14]. Many inpatients with those mental disorders can exhibit impulsiveness. The characteristics of this impulsive violence involves a reactive or emotional response with a loss of behavioral control, coupled with a failure to consider consequences [50]. This supported the rationality of this study. Therefore, by using our proposed IPEQs, medical workers in the non-psychiatric department could collect the necessary information to assess six mental health disorders of an inpatient, and thereby better prepare themselves to provide individual medical care accordingly. The communication barriers between patients and medical workers will be reduced, and a friendly patient-doctor relationship will be rebuilt up.

There are several limitations that we will addressed in future studies. First, all participants were from only one general hospital in Taiyuan, Shanxi Province. The proposed IPEQs should be validated in non-psychiatric departments and data should be collected for further evaluation of the tool. Second, we selected six mental disorders that were implicated to have strong association with violence. Adding other violence-related mental health disorders (e.g. dissocial personality disorder) for testing may improve the accuracy of the IPEQs. Third, we did not use golden standard in the cut-off study. A main future research would be to recruit participants with psychiatrists’ diagnoses, which will be suggested as golden standard to calculate sensitivity and specificity. In that way, we can improve the cut-off setting for IPEQs. Forth, our study only used three statistical methods to select items. Other statistical methods (e.g. Velicer’s minimum average partial (MAP) Test combined with the parallel analysis in EFA [51]) may help in the item evaluation and selection.

## Conclusion

We developed and evaluated two IPEQs that demonstrated satisfactory reliability and validity. By using clinical symptoms or personality traits associated with violence-related mental health disorders, patients with violent tendencies would be screened. However, further studies are needed in different participants to revise and improve IPEQs. This initial study guaranteed further validation of the two proposed IPEQs in non-psychiatric departments of the general hospitals in China.

## Supplementary information

**Additional file 1: Supplementary Table 1.** The final version of IPEQ-1. **Supplementary Table 2.** The final version of IPEQ-2. **Supplementary Fig. 1.** Test information and measurement error and matrix plot of item characteristic curves obtained from the 18 (second version) items of IPEQ-1 using IRT. **Supplementary Fig. 2.** Test information and measurement error and matrix plot of item characteristic curves obtained from the 21 (second version) items of IPEQ-2 using IRT

## Data Availability

The dataset used and analyzed during the current study is available from the corresponding author on reasonable request.

## Abbreviations

IPEQ-1: Inpatient psychological experience questionnaire-1
IPEQ-2: Inpatient psychological experience questionnaire-2
PPD: Paranoid personality disorder
EUPD: Emotionally unstable personality disorder
HPD: Histrionic personality disorder
ICD-10: International statistical classification of diseases and related health problems 10th revision
GAD-7: Generalized anxiety disorder-7
PHQ-9: Patient health questionnaire-9 items
PDQ-4 +: Personality diagnostic questionnaire-4+.
PCC: Pearson’s correlation coefficient
EFA: Exploratory factor analysis
IRT: Item response theory
CFA: Confirmatory factor analysis
RMSEA: Root mean square error of approximation
GFI: Goodness-of-fit index
AGFI: Adjusted goodness of fit index
NFI: Normed fit index
NNFI: Non-normed fit index
CFI: Comparative fit index
