# Supplementary material for: Development of two psychological experience questionnaires for screening violence-related mental health disorders of non-psychiatric inpatients
Source: Health Qual Life Outcomes. 2020 May 25;18:151. doi: 10.1186/s12955-020-01399-9 (PMC7249322; doi:10.1186/s12955-020-01399-9)
Supplement: Supplementary file 1 — Additional file 1: Supplementary Table 1. The final version of IPEQ-1. Supplementary Table 2. The final version of IPEQ-2. Supplementary Fig. 1. Test information and measurement error and matrix plot of item characteristic curves obtained from the 18 (second version) items of IPEQ-1 using IRT. Supplementary Fig. 2. Test information and measurement error and matrix plot of item characteristic curves obtained from the 21 (second version) items of IPEQ-2 using IRT [file 12955_2020_1399_MOESM1_ESM.docx]

**Supplementary Table 1** The final version of IPEQ-1

| How often they had experienced the feelings described in the questionnaire over the last 2 weeks? | Never | Occasionally | Some of the time | Most of the time | Nearly all the time |
| --- | --- | --- | --- | --- | --- |
| 1. Felt worried or nervous? | 0 | 1 | 2 | 3 | 4 |
| 2. Felt afraid for no reason? | 0 | 1 | 2 | 3 | 4 |
| 3. Felt uneasy? | 0 | 1 | 2 | 3 | 4 |
| 4. Felt too unstable to calm down? | 0 | 1 | 2 | 3 | 4 |
| 5. Felt so depressed that nothing could make you happy? | 0 | 1 | 2 | 3 | 4 |
| 6. Felt uninterested in any part of your daily routine? | 0 | 1 | 2 | 3 | 4 |
| 7. Felt unable to proceed with daily tasks? | 0 | 1 | 2 | 3 | 4 |
| 8. Felt tired and listless? | 0 | 1 | 2 | 3 | 4 |
| 9. Felt purposeless in life? | 0 | 1 | 2 | 3 | 4 |
| 10. Felt death would be a release? | 0 | 1 | 2 | 3 | 4 |
| 11. Had thoughts of ending your life? | 0 | 1 | 2 | 3 | 4 |
| 12. Self-harmed or performed suicidal behavior? | 0 | 1 | 2 | 3 | 4 |

All items are used 5-point Likert scale strategy. (0 = Never, 1= Occasionally, 2 = Some of the time, 3 = Most of the time, 4 = Nearly all the time)

**Supplementary Table 2** The final version of IPEQ-2

| Please choose the best option on the questionnaire depending on your actual thoughts from the age of 18 years to the present. | Yes | No |
| --- | --- | --- |
| 1. You are vigilant in dealing with people to prevent them from using or harming you? | 1 | 0 |
| 2. You’re always unsatisfied with what people around you are doing? | 1 | 0 |
| 3. You think that others will take advantage of you or try to deceive you? | 1 | 0 |
| 4. You hesitate to trust others or cannot trust others? | 1 | 0 |
| 5. You talk and do things without thinking? | 1 | 0 |
| 6. It’s easy to do things recklessly and fail to control your actions well? | 1 | 0 |
| 7. You often do something reckless? | 1 | 0 |
| 8. You can’t handle relationships with others well? | 1 | 0 |
| 9. In order to attract people’s attention, you may pretend to surprise at small matters? | 1 | 0 |
| 10. You consider yourself more attractive than others? | 1 | 0 |
| 11. You draw the attention of others by your clothes or by certain behaviors? | 1 | 0 |
| 12. You care about your appearance? | 1 | 0 |

All items are used 2-point strategy. (1 = Yes；0 = No)

**Supplementary Fig. 1** Test information and measurement error and matrix plot of item characteristic curves obtained from the 18 (second version) items of IPEQ-1 using IRT

**Supplementary Fig. 2** Test information and measurement error and matrix plot of item characteristic curves obtained from the 21 (second version) items of IPEQ-2 using IRT
